# Prevalence of red panda amdoparvovirus infection in European zoos

**DOI:** 10.3389/fvets.2023.1276248

**Published:** 2023-10-25

**Authors:** Urška Kuhar, Oldřich Tomášek, Endre Sós, Jana Mede, Marjan Kastelic, Nuša Jež, Michaela Petrikova, Trine Hammer Jensen, Charles Everett Alex, Urška Jamnikar-Ciglenecki, Pavel Kvapil

**Affiliations:** ^1^Institute of Microbiology and Parasitology, Veterinary Faculty, University of Ljubljana, Ljubljana, Slovenia; ^2^Institute of Vertebrate Biology, Czech Academy of Sciences, Brno, Czechia; ^3^Budapest Zoo and Botanical Garden/Budapest University of Veterinary Science, Budapest, Hungary; ^4^University of Veterinary Medicine Budapest, Budapest, Hungary; ^5^Veterinary Department of the Ljubljana Zoo, Ljubljana, Slovenia; ^6^Avian and Exotic Animal Clinic, Faculty of Veterinary Medicine, University of Veterinary and Pharmaceutical Sciences, Brno, Czechia; ^7^Aalborg Zoo/Aalborg University, Aalborg, Denmark; ^8^Department of Pathology, Microbiology, and Immunology, School of Veterinary Medicine, University of California–Davis, Davis, CA, United States; ^9^Zoological Health Program, Wildlife Conservation Society, Bronx Zoo, Bronx, NY, United States; ^10^Institute of Food Safety, Feed, and Environment, Veterinary Faculty, University of Ljubljana, Ljubljana, Slovenia

**Keywords:** *Ailurus fulgens*, red panda, amdoparvovirus, RPAV, zoo

## Abstract

Red panda amdoparvovirus (RPAV) was first described in captive red pandas (*Ailurus fulgens*) at a zoo in the United States in 2018. Subsequently, the prevalence of infection in zoos in the United States was reported to be 50%; however, RPAV prevalence outside the United States remains unstudied. This study was conducted to investigate the prevalence of RPAV in 134 red pandas from zoos in Europe. Overall, RPAV was detected with PCR in 21 of 62 zoos (33.9%), and the virus prevalence among individuals was estimated to be 24.2% (95% confidence interval, 17.4%–32.0%). Remarkably, adult females tested positive for RPAV more frequently than adult males. Zoos where RPAV was detected reported a significantly higher occurrence of alopecia (and clinical signs in general), whereas other commonly reported problems (fecal disorders and dental disease) showed no difference. A repeated pooled sampling of two positive individuals further showed that RPAV excretion in feces is intermittent, with the viral DNA being only detected on 8 out of 14 sampling days. The intermittent nature of excretion implies that RPAV prevalence may be higher than the estimated value.

## 1. Introduction

Amdoparvovirus is a genus within the *Parvoviridae* family, a group of small, nonenveloped viruses with a linear, single-stranded DNA (ssDNA) genome (1). Some of the most important viruses causing clinical diseases with often fatal outcomes in domestic and wild carnivores (2–4) belong to the *Parvoviridae* family, such as canine parvovirus (CPV), feline panleukopenia virus (FPV), or Aleutian mink disease virus (AMDV). AMDV was the first amdoparvovirus described and is the best-characterized amdoparvovirus, whereas knowledge of the clinical signs, pathogenesis, and epidemiology in other species in this genus is rather limited. AMDV causes an immune complex-associated progressive syndrome known as Aleutian mink disease, a common disease in farmed mink that is often fatal and causes major economic losses (5–8). Other amdoparvoviruses have been described in gray fox (gray fox amdovirus, GFAV), raccoon dog and fox (raccoon dog and fox amdoparvovirus, RFAV), and skunk (skunk amdoparvovirus, SKAV) (9–11). The most recently recognized member of this genus is red panda amdoparvovirus (RPAV) (4, 12).

The red panda (*Ailurus fulgens*) is a small omnivore of the order Carnivora, native to the eastern Himalayas and southwestern China. Its population in the wild is estimated to be around 10,000 individuals (13). The conservation status of the red panda on the IUCN Red List was changed from vulnerable to endangered in 2015 due to a 50% decline in wild red panda populations over the past three generations caused by habitat loss and fragmentation, hunting, and poaching (14). Given the rapid population decline, an international studbook for the species was established in 1979 to monitor the population under human care. In 1985, a European Endangered Species Programme (EEP) was established and, from then on, the captive red panda population began to increase due to improved breeding techniques and husbandry. From 1985, when the total number of individuals was 53, the European red panda population increased to 416 animals housed in 188 facilities by the end of 2020 (15, 16).

One of the important tasks in maintaining sustainable populations, both in captivity and in the wild, is to minimize the risk of infectious diseases (17, 18). However, there is little information on the prevalence and significance of infectious diseases, especially viral diseases, in red pandas. Red pandas are known to be susceptible to canine distemper virus (CDV) (19–21) and have also been diagnosed with rabies virus infection (22). In China, a novel parvovirus, similar to canine parvovirus CPV-2a, was isolated from a red panda displaying no clinical abnormalities (23). Reports of other viral infections in red pandas are rare (21).

In 2018, the first RPAV infection was reported in captive red pandas at a zoo in the United States (12). The combination of histopathological findings and *in situ* hybridization (ISH) for virus within lesions provided early evidence of the pathogenicity of this virus (12). That report led us to test for a suspected RPAV infection in a red panda from the Ljubljana Zoo, which died in 2019. The results of PCR performed on tissue samples and sequencing of the PCR product confirmed infection with RPAV in the deceased animal (24). This was the first case of RPAV infection in a European zoo collection known to the authors of this study. The cause of death of the red panda from the Ljubljana Zoo, which was attributed to RPAV infection, was further investigated using ISH, and acute myocarditis and encephalitis were confirmed as the primary cause of death in the infected animal (unpublished results). Neurological signs such as ataxia and discoordination were observed 18 months before death. The clinical signs described resolved spontaneously (24).

Recently, a study of RPAV infection in zoo-housed red pandas in the United States revealed a high prevalence (50%) of infection, constant virus shedding in feces, and high genetic diversity of the virus (25). To date, no studies have been published on the detection of RPAV and the prevalence of infection in red pandas in European zoos or elsewhere outside the United States. Moreover, clinical consequences and disease manifestations are not well understood. Therefore, this study was conducted to investigate the prevalence of RPAV and its potential association with clinical signs in red pandas in European zoos.

## 2. Materials and methods

### 2.1. Sample collection

Zoos from the EEP (European *Ex-situ* Program) housing red pandas were invited to participate in the study. There are 188 zoos on the EEP list. A total of 62 of 188 listed zoos from Europe (including one zoo from the French region of Guadeloupe in the Caribbean) and one from Israel participated by submitting samples. Each participating zoo also provided epidemiological information, including age (birth date), sex, and date of sampling for each animal.

A total of 113 samples from 134 red pandas were collected in 2020–2022. Individual samples were obtained from 95 animals consisting of 90 fecal samples, four tissue samples tested as a pool, and a sample of paraffin-embedded formalin-fixed tissues from one animal. In addition, this sample set was supplemented by 18 pooled samples from 39 animals. In cases of positive results in pooled samples, zoos were asked to send samples from individual animals. A total of 19 pooled samples were initially sent for testing, of which three were positive. One of these zoos consequently sent samples from individual animals (which are included in the final set of 95 individual samples described above), and so the final pooled sample set included pooled samples from 18 zoos. Samples were frozen at −20°C until shipment to the laboratory for PCR analysis and frozen at −80°C until further processing. All laboratory work was performed at the Institute for Microbiology and Parasitology, Veterinary Faculty, University of Ljubljana, Slovenia.

To evaluate whether RPAV is shed continuously in feces of infected animals, 14 pooled feces samples from the Ljubljana Zoo red panda (*n* = 2) group were collected at approximately 2-day intervals over 1 month.

### 2.2. Clinical signs in the zoo survey

Concurrent with fecal sampling, the 113 EPP zoos were asked to complete an on-line survey focusing on detection of clinical signs in red pandas. All zoos included were contacted by email and 54 of the 62 zoos that sent samples completed the survey.

### 2.3. Sample processing and PCR

Five percent suspensions of fecal samples and 10% suspensions of tissue samples were prepared using RPMI medium 1,640 (Thermo Fisher Scientific, Carlsbad, CA, United States). The suspensions were homogenized and centrifuged at 2,000 × *g* for 10 min. The supernatant was used for DNA extraction using the MagMAX™ CORE Nucleic Acid Purification Kit on the KingFisher Flex System (Thermo Scientific) according to the manufacturer’s instructions. DNA was extracted from one animal from tissues embedded in paraffin and fixed in formalin using the DNeasy Blood & Tissue Kit (Qiagen, Germany) according to the manufacturer’s instructions.

For the detection of RPAV DNA, a RPAV-specific PCR with forward primer RPAmdoNS_F2 (5′-CGCCAAAACCAACCGACCAA-3′) and reverse primer RPAmdoNS_R2 (5′- AACACGCCCTTAGCTGTGCTT-3′) (12) was used, which amplifies a 154-nucleotide segment of the nonstructural (NS1) region of the RPAV genome. The PCR products were visualized with the QIAxcel Capillary Electrophoresis System (Qiagen, Germany).

### 2.4. Statistical analysis

Statistical analysis was performed with R 4.1.2 software (26). RPAV prevalence was estimated using the function PoolPrev from the R package PoolTestR (27), which allows prevalence estimation from a combination of individual and pooled samples. The maximum likelihood method of estimation was used.

To examine the possible presence of sex- and age-related differences in RPAV infection probability, generalized mixed-effect models implemented in the R package lme4 (28) were employed. Specifically, logistic mixed-effect regression was fitted to the subset of individual samples from positive zoos. Infection status was fitted as the response variable, sex and age were fitted as fixed predictors, and zoo identity was included as a random grouping factor to control for the non-independence of samples obtained from the same zoo. Negative zoos were excluded from this analysis because, due to the absence of the virus, these zoos provide no information regarding the between-group differences in the probability of becoming infected. For this reason, inclusion of the negative zoos could dilute and obscure the potential existing differences. To demonstrate this effect, we also repeated the analysis using individual samples from all zoos.

The possible association between RPAV infection with clinically apparent health problems was analyzed using logistic regression. The presence of RPAV and the occurrence of clinical signs at the zoo level were used in this analysis, given that many zoos only provided the general occurrence of clinical signs in their group of red pandas instead of the individual-level data. Four models were built to test for the association of RPAV with either the occurrence of any clinical signs or the occurrence of one of the three most commonly reported problems (i.e., alopecia, diarrhea or mucoid feces, and dental disease). In each model, the occurrence of the target clinical signs (any or one of the three most common signs listed above) in the zoo was fitted as a binary response variable (signs present or not present). The presence or absence of RPAV in the zoo was fitted as the only predictor.

## 3. Results

### 3.1. RPAV prevalence

**Figure 1:**
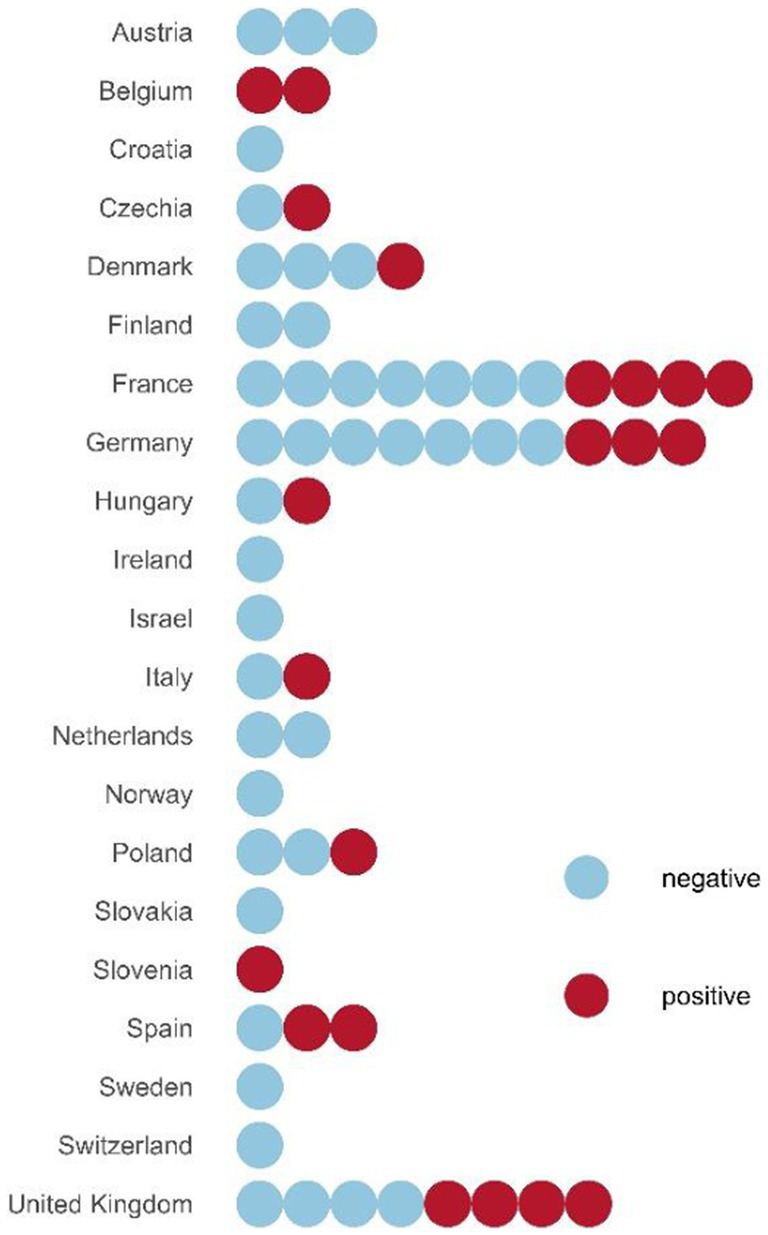
RPAV positive and RPAV negative zoos by country. Dots represent individual zoos. Negative zoos are those with no RPAV positive samples, whereas positive zoos are those with at least one sample tested positive for RPAV.

**Figure 2:**
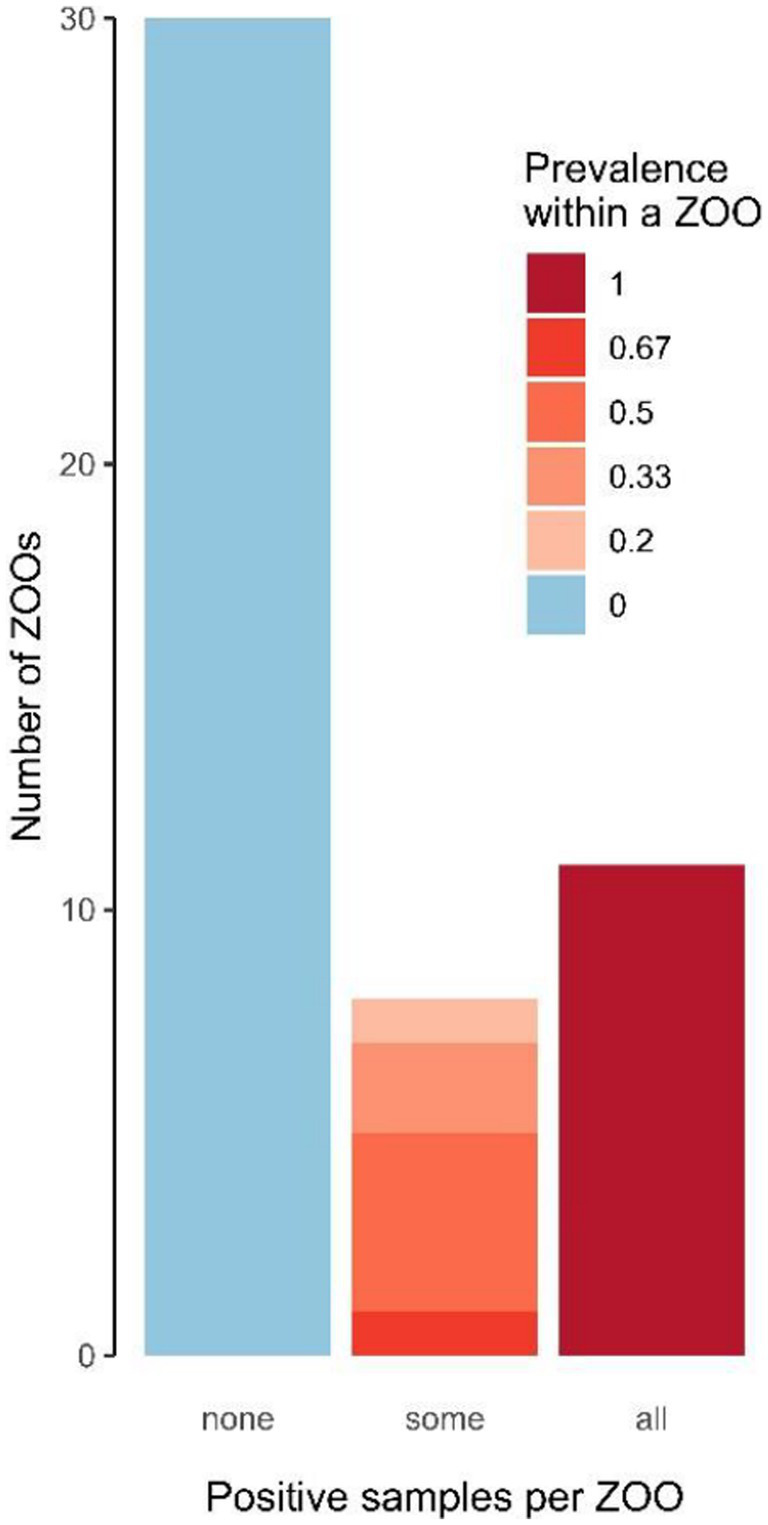
Numbers of zoos according to the within-zoo proportion of RPAV positive samples.

Animals under the age of 1.5 years at the time of sampling were considered juvenile (*n* = 22), whereas older ones were considered adult (*n* = 112). Overall, RPAV DNA was detected in 21 out of 62 zoos (33.9%; Figure 1). Using regression modeling implemented in the R package PoolTestR with the entire data set combining individual and pooled samples, the RPAV prevalence among individuals was estimated to be 24.2% with the 95% confidence interval ranging from 17.4 to 32.0%. In 11 out of 49 zoos providing individual samples, RPAV DNA was detected in all animals tested (Figure 2), including four zoos that housed only one animal (two of them included as archival tissue samples from dead animals). In another eight zoos, RPAV was detected only in a proportion of tested animals. Considering only tissue samples from dead animals examined in this study, four out of five animals were positive for RPAV DNA, including one animal that died in 1996.

### 3.2. Sex- and age-related differences in RPAV prevalence

The numbers and proportions of positive animals in a set of individual samples broken down by sex and age categories are shown in Table 1. Statistical analysis of sex-related differences in probability of RPAV detection performed in the subset of zoos where at least one positive sample was detected (*n* = 44 individual samples from 19 zoos) revealed that the odds of testing positive for RPAV were 5.2 times (95% confidence interval: 1.2–31.7) higher in females (mixed-effect logistic regression: estimate = 1.66, *SE* = 0.79, *z* = 2.09, *p* = 0.037). The analysis including only adult individuals (*n* = 38 individuals from 19 zoos) and excluding the age category from the model produced very similar results (estimate = 2.17, *SE* = 1.03, *z* = 2.11, *p* = 0.035), indicating that adult females had 8.8 times (95% confidence interval: 1.5–123.4) higher odds of testing positive for RPAV compared to adult males. To demonstrate the dilution effect of including animals from the RPAV-negative zoos, we repeated the analysis using individual samples (*n* = 95) from all the 60 zoos, which indeed revealed no significant differences between sexes (estimate = 1.09, *SE* = 0.87, *z* = 1.26, *p* = 0.21). Juveniles showed no significant difference in RPAV positivity compared to adults (estimate = −0.55, SE = 1.04, *z* = −0.53, *p* = 0.59).

**Table 1:** Numbers and proportions of positive animals based on a set of individual samples grouped by sex and age categories.

		All zoos			Positive zoos only		
Age category	Sex	Positive	Total	Percent	Positive	Total	Percent
Adult	Female	19	48	39.6	19	22	86.4
Adult	Male	8	33	24.2	8	16	50.0
Juvenile	Female	1	8	12.5	1	2	50.0
Juvenile	Male	2	6	33.3	2	4	50.0

Numbers are shown from a subset of zoos with at least one positive sample, as well as all zoos included in the study.

### 3.3. RPAV shedding persistence

The RPAV shedding persistence in feces over a 1-month period was evaluated using pooled samples from the Ljubljana Zoo group. The results showed that only eight out of 14 samples were positive (Table 2), indicating that excretion of RPAV in the feces is intermittent.

**Table 2:** Results of the PCR detection of RPAV over a 1-month period at the Ljubljana Zoo.

Date of sample collection	PCR result
April 5th, 2021	**Positive**
April 7th, 2021	Negative
April 9th, 2021	Negative
April 12th, 2021	Negative
April 14th, 2021	**Positive**
April 16th, 2021	Negative
April 19th, 2021	**Positive**
April 21st, 2021	**Positive**
April 23rd, 2021	**Positive**
April 26th, 2021	Negative
April 28th, 2021	Negative
April 30th, 2021	Negative
May 3rd, 2021	Negative
May 5th, 2021	**Positive**

### 3.4. Zoo-level co-occurrence of RPAV and clinical signs

The zoos where RPAV was detected were 3.3 times (95% confidence interval: 1.04–11.17) more likely to report the occurrence of any clinical signs compared to the RPAV-negative zoos (logistic regression: estimate = 1.20, *SE* = 0.60, *z* = 2.01, *p* = 0.045). The three most commonly reported clinical signs were alopecia, diarrhea or mucoid feces, and dental disease. Out of these three health disorders, alopecia was 6.4 times (95% confidence interval:1.5–33.7) more likely to be reported by the RPAV-positive zoos (estimate = 1.86, *SE* = 0.77, *z* = 2.42, *p* = 0.016), whereas neither digestive disorders (estimate = 0.41, *SE* = 0.82, *z* = 0.49, *p* = 0.62) nor dental disease (estimate = −0.49, *SE* = 1.19, *z* = −0.41, *p* = 0.68) showed an association with RPAV occurrence at the zoo level.

## 4. Discussion

This study is the first to investigate the presence of RPAV in red pandas from zoos in Europe. Based on the previous reports that RPAV is present in the feces of infected red pandas (12, 25), this study primarily focused on the detection of RPAV DNA in fecal samples. In addition, tissue samples from dead red pandas were included to expand the data set. To date, there has only been one study of the prevalence of RPAV in red pandas conducted in zoos in the United States (25), which included 104 animals from 37 zoos. That study detected viral DNA in 52/104 samples (50.0%), which is higher than the prevalence estimated for European zoos in our study (24.2%). Based on comparable sample sizes, 104 animals in the United States versus 134 animals in Europe, viral DNA was detected in 50.2% of the samples in that study compared to 24.2% in our current study. In addition to the higher prevalence, there is also a higher percentage of positive zoos in the United States (67.6%) compared to Europe (33.9%). The causes of the difference in RPAV prevalence between the United States and Europe are unknown. Factors that may drive the potential differences in the infection rates could include differences in zoo management, animal husbandry, or the time when RPAV was introduced to the respective continents. The different subspecies kept in European and American zoos may also play a role in the different prevalence of RPAV; European zoos house only *Ailurus fulgens fulgens* while United states zoos house both *A. f. styani* and *A. f. fulgens*.

The study by Alex et al. (25) reported that the virus was shed continuously in the feces in all but two cases of infected animals and could be detected there for more than 4.5 years. In our study, we examined the persistence of virus excretion in feces over a period of 1 month. In contrast to the previous study, our results showed intermittent RPAV excretion, with the viral DNA being only detected on 8 out of 14 sampling days. Such intermittent viral excretion suggests that the actual prevalence may be somewhat higher than the value estimated in our study because, the sampling only occurred at one time point in most zoos, which may have resulted in the occurrence of false negative results. Another potential source of false negative results could be the high sequence diversity among RPAV strains, which could result in positive cases being missed due to mutations in the binding sites of the primers used in PCR (12). Our study suggests that the virus does not shed constantly, as Alex et al. (25) suggested; however, the small number of samples and short monitoring period as well as different methodology in sample collection can make a difference. Virus shedding persistence needs to be confirmed in further studies.

In contrast to the prevalence study in the United States (12), which reported no significant differences in infection status between sexes, our data showed that adult females were significantly more likely to test positive for RPAV compared to adult males. Such a discrepancy between the studies could possibly be explained by the differences in the design of statistical analysis. Given that only zoos where the virus is present are informative regarding the between-group differences in the probability of being infected, we excluded non-informative negative zoos from this analysis. In contrast, Alex et al. (25) analyzed sex differences across all the zoos, and the inclusion of negative zoos may have obscured the effect of sex as demonstrated in our study. Nevertheless, further studies are needed to investigate whether potential differences in virus dispersion, husbandry, or animal behavior could contribute to the difference in sex-related infection probability between American and European zoos. Moreover, the potential differences in resistance to RPAV between male and female red pandas represent another interesting topic for future research.

In agreement with the study by Alex et al. (25), our study found no significant difference between juvenile and adult animals. However, the different statistical design of the American prevalence study and the small sample size of juvenile animals included in our study prevent any robust conclusion about age-related differences in RPAV prevalence.

Although RPAV was not described until 2018, our study provides evidence that the virus was already present in captive red pandas in Europe in the 1990s since we detected RPAV DNA in an archival sample from a red panda that died in 1996 in a zoo in France. The virus may have remained undetected because the infection was subclinical or because most infected animals had mild or nonspecific clinical signs and specific diagnostic methods were not available. Detection of this novel amdoparvovirus was only made possible by modern high-throughput sequencing technology, allowing detection of pathogens without prior information about its type, genome structure, and infection symptoms (12).

The survey of clinical signs, which was simultaneously conveyed with the sample collection, revealed that the most common health problems reported in zoo-housed red pandas include alopecia, diarrhea or mucoid feces, and dental disease. Interestingly, the zoos where RPAV was detected were more likely to report the occurrence of any clinical signs in general. This association was probably likely driven by alopecia, which was significantly more often present in RPAV-positive zoos. In contrast, no such association was observed with fecal or dental disorders. These results suggest that RPAV could have deleterious health consequences in the RPAV-infected red pandas. Alopecia in particular seems to be a candidate disorder potentially associated with RPAV infection. Nevertheless, it must be borne in mind that our analysis was only carried out at the zoo level due to the limitations of our survey-based clinical data set. More detailed studies including individual data on clinical signs are needed to draw more robust conclusions about the link between RPAV infection and alopecia or other health disorders.

Our study as well as the recent study by Alex et al. (25) showed that the RPAV infection is common in captive red pandas in both American and European zoos. As a part of the conservation management of red pandas, individuals are constantly translocated among zoos, and undetected pathogens could thereby be transmitted from one zoo to another. Although not much is known about the pathogenicity of RPAV, the infection status should be considered when planning transfers of red pandas to avoid introducing the virus into an infection-free group. The presence and prevalence of RPAV in wild red pandas also need to be evaluated to avoid the potential risk of bringing this infectious agent into the potentially naïve wild population. Persistent latent or subclinical infection in captive red pandas has likely led to underdiagnosing this virus during necropsies and the underappreciation of lesions associated with RPAV that may contribute to the death of animals. Retrospective studies of stored samples in both the United States and Europe as well as prospective development of improved necropsy protocols should be put in place to discover subtle pathologies and lesions caused by the virus. Another step forward to be considered is future control of this disease if pathogenicity is proved to be considerable in this species. Nonetheless, pre-shipment testing is recommended covering an extended period to detect intermittent shedding virus. Due to the very high prevalence of RPAV, maintaining disease-free populations may not be a viable option. Thus, in the long-term development of vaccines may offer the best solution for minimizing disease and spread of the virus to naive collections of Red Pandas.

## Data availability statement

The original contributions presented in the study are included in the article/Supplementary material, further inquiries can be directed to the corresponding author.

## Ethics statement

The animal study was approved by all procedures involving animals were approved by the National Ethical Committee and the Administration of the Republic of Slovenia for Food Safety, Veterinary and Plant Protection (Permit number 34401-7-2016-5, 31 January 2017). The study was conducted in accordance with the local legislation and institutional requirements.

## Author contributions

UK: Conceptualization, Investigation, Methodology, Resources, Supervision, Writing – original draft. OT: Data curation, Formal analysis, Methodology, Project administration, Resources, Software, Visualization, Writing – original draft. ES: Methodology, Writing – review & editing. JM: Conceptualization, Project administration, Software, Software. MK: Data curation, Funding acquisition, Resources, Validation, Visualization, Writing – review & editing. NJ: Data curation, Validation, Visualization, Writing – review & editing. MP: Investigation, Software, Writing – review & editing. TJ: Validation, Writing – review & editing. CA: Conceptualization, Writing – review & editing. UJ-C: Investigation, Validation, Writing – review & editing. PK: Conceptualization, Data curation, Formal analysis, Funding acquisition, Methodology, Resources, Supervision, Writing – original draft.
