# Supplementary material for: Prevalence of red panda amdoparvovirus infection in European zoos
Source: Front Vet Sci. 2023 Oct 25;10:1276248. doi: 10.3389/fvets.2023.1276248 (PMC10634534; doi:10.3389/fvets.2023.1276248)
Supplement: Supplementary file 1 [file Data_Sheet_1.PDF]

ZOO by country	CurrentProblems	Alopecia	FaecalDisorders	DentalDisease	Positive
Spain	0	0	0	0	1
Germany	1	1	0	0	1
Czechia	1	1	0	0	1
Germany	0	0	0	0	1
Denmark	1	1	0	0	1
Poland	0	0	0	0	1
United Kingdom	1	0	0	1	1
France	0	0	0	0	1
Germany	0	0	0	0	1
France	1	0	1	0	1
France	0	0	0	0	1
Spain	1	1	0	0	1
Slovenia	1	1	0	0	1
United Kingdom	1	1	1	0	1
United Kingdom	1	0	1	0	1
Belgium	0	0	0	0	1
France	0	0	0	0	1
United Kingdom	0	0	0	0	1
Hungary	1	1	0	0	1
Italy	0	0	0	0	0
Germany	1	1	0	0	0
Finland	1	0	1	1	0
Switzerland	1	1	1	0	0
France	0	0	0	0	0
Germany	0	0	0	0	0
Norway	1	0	0	1	0
Austria	0	0	0	0	0
Germany	0	0	0	0	0
France	0	0	0	0	0
Denmark	0	0	0	0	0
Slovakia	0	0	0	0	0
France	1	0	0	0	0
Hungary	0	0	0	0	0
Germany	0	0	0	0	0
France	0	0	0	0	0
Austria	0	0	0	0	0
Poland	1	0	1	0	0
Netherlands	0	0	0	0	0
Ireland	0	0	0	0	0
Spain	0	0	0	0	0
Denmark	0	0	0	0	0
Germany	1	0	1	0	0
United Kingdom	0	0	0	0	0
United Kingdom	0	0	0	0	0
Austria	0	0	0	0	0
Poland	0	0	0	0	0
Germany	0	0	0	0	0
France	1	1	0	0	0
Finland	1	0	0	1	0
France	0	0	0	0	0

France	0	0	0	0	0
Israel	0	0	0	0	0
United Kingdom	0	0	0	0	0
Czechia	0	0	0	0	0
Sweden	0	0	0	0	0
