# Supplementary material for: Prevalence of red panda amdoparvovirus infection in European zoos
Source: Front Vet Sci. 2023 Oct 25;10:1276248. doi: 10.3389/fvets.2023.1276248 (PMC10634534; doi:10.3389/fvets.2023.1276248)
Supplement: Supplementary file 2 [file Data_Sheet_2.PDF]

ZOO Number	Country	Date of birth (death) or age	Adult (A) / Juvenile (J)	Sex	Date of sampling	Sample type	PCR result
1	Italy	10.06.2013	A	Male	25.05.2020	faeces	Neg
		31.07.2014	A	Female	25.05.2020	faeces	Neg
2	Germany	27.06.2015	A	Male	26.05.2020	faeces	Neg
		23.06.2014	A	Female	26.05.2020	faeces	Pos
3	Denmark	19.07.2015	A	Female	28.05.2020	faeces (pool)	Neg
		22.06.2016	A	Male	28.05.2020		
4	Germany	14.07.2015	A	Male	26.05.2020	faeces	Neg
		4.07.2014	A	Female	26.05.2020	faeces	Neg
		26.08.2012	A	Male	26.05.2020	faeces	Neg
		14.07.2013	A	Female	26.05.2020	faeces	Pos
		28.06.2021	J	Male	16.03.2022	faeces	Neg
5	Germany	9.07.2012	A	Female	2.06.2020	faeces (pool)	Neg
		22.06.2016	A	Male	2.06.2020		
		10.07.2019	J	Female	2.06.2020	faeces	Neg
6	France	4.07.2008	A	Female	2.06.2020	faeces	Pos
		6.07.2019	J	Male	2.06.2020	faeces	Neg
		3Y (died 1996)	A	Male	NA	tissues	Pos
7	United Kingdom	11Y 9M (died 2020)	A	Male	2.06.2020	faeces	Pos
8	Germany	6.06.2014	A	Male	4.06.2020	faeces	Neg
		22.07.2013	A	Female	4.06.2020	faeces	Pos
9	France	28.06.2015	A	Female	28.05.2020	faeces	Neg
10	Czech Republic	2.07.2010	A	Male	5.06.2020	faeces	Neg
		5.06.2012	A	Female	21.02.2022	faeces (pool)	Neg
		29.06.2021	J	Male	21.02.2022		
		29.06.2021	J	Female	17.03.2022	faeces	Neg
		28.06.2019	J	Male	5.06.2020	faeces	Pos
11	Denmark	20.06.2014	A	Male	8.06.2020	faeces	Pos
		25.06.2016	A	Female	9.06.2020	faeces	Pos
12	Finland	3.07.2014	A	Female	1.06.2020	faeces	Neg
		19.06.2014	A	Female	1.06.2020	faeces	Neg
13	Slovenia	8Y (died 2019)	A	Female	NA	tissues	Pos
		18.06.2018	A	Female	26.04.2020	faeces	Pos
		6.06.2012	A	Male	26.04.2020	faeces	Pos
14	Switzerland	7.07.2009	A	Female	15.06.2020	faeces	Neg
		1.06.2009	A	Male	15.06.2020	faeces	Neg
15	France	27.06.2004	A	Female	15.06.2020	faeces	Neg
16	Germany	3.07.2016	A	Male	14.,15.6.2020	faeces	Neg
		13.07.2009	A	Female	14.,15.6.2021	faeces	Neg
17	Norway	27.06.2012	A	Male	28.05.2020	faeces (pool)	Neg
		23.06.2018	A	Female	28.05.2020		
		15.06.2013	A	Male	16.06.2020 and 25.12.2020-8.1.2021	faeces	Neg

18	France	4.07.2012	A	Female	18.-30.1.2021	faeces	Pos
		5.07.2019	J	Female	3.-17.2.2021	faeces	Neg
		5.07.2019 (8.9.2020 died)	J	Female	NA	tissues	Pos
19	Austria	11.06.2012	A	Male	15.06.2020	faeces	Neg
		18.06.2012	A	Female	15.06.2020	faeces	Neg
20	Germany	11.06.2019	J	Male	16.06.2020	faeces (pool)	Neg
		19.07.2013	A	Male	16.06.2020		
		28.06.2015	A	Female	16.06.2020		
21	Belgium	18.08.2010	A	Female	22.06.2020	faeces	Neg
		2.07.2018	A	Male	22.06.2020	faeces	Pos
22	France	6.03.2015	A	Female	21.06.2020	faeces	Neg
		6.03.2015	A	Female	21.06.2020	faeces (pool)	Neg
		12.10.2017	A	Female	21.06.2020		
23	Netherlands	17.06.2019	J	Female	15.06.2020	faeces	Neg
		11.06.2019	J	Female	15.06.2020	faeces	Neg
24	Denmark	29.06.2015	A	Female	22.06.2020	faeces	Neg
		29.06.2015	A	Female	22.06.2020	faeces	Neg
25	Spain	3.07.2016	A	Female	23.06.2020	faeces	Pos
26	Slovakia	25.06.2010	A	Female	22.06.2020	faeces	Neg
		23.06.2010	A	Male	22.06.2020	faeces	Neg
27	France	16.06.2014	A	Female	19.06.2020	faeces	Pos
28	France	9.06.2019	J	Female	12.06.2020	faeces	Neg
		26.06.2007	A	Male	22.06.2020	faeces	Neg
		7.06.2016	A	Female	22.06.2020	faeces	Neg
29	Hungary	28.07.2013	A	Female	26.06.2020	faeces	Neg
		28.07.2013	A	Female	26.06.2020	faeces	Neg
		4.07.2019	J	Male	26.06.2020	faeces	Neg
30	Germany	21.06.2010	A	Male	30.06.2020	faeces (pool)	Neg
		4.07.2011	A	Female	30.06.2020		
31	Spain	30.06.2010	A	Female	6., 7.7.2020	faeces	Pos
		30.06.2006	A	Male	6.,7.7.2020	faeces	Pos
		1.07.2009	A	Female	6.,7.7.2020	faeces	Pos
32	France	28.06.2015	A	Female	7.07.2020	faeces	Neg
33	Croatia	27.06.2012	A	Male	9.07.2020	faeces	Neg
		25.06.2014	A	Female	9.07.2020	faeces (pool)	Neg
		4.07.2019	J	Male	9.07.2020		
		3.07.2019	J	Female	9.07.2020		
34	Austria	26.06.2015	A	Male	7.07.2020	faeces	Neg
		11.06.2015	A	Female	7.07.2020	faeces	Neg
35	Poland	23.06.2018	A	Male	12.07.2020	faeces	Neg
		23.06.2018	A	Male	12.07.2020	faeces	Neg
36	Poland	30.06.2015	A	Male	15.05.2020	faeces	Neg
		19.07.2015	A	Male	13.07.2020	faeces	Neg
		21.06.2013	A	Female	13.07.2020	faeces	Pos

37	United Kingdom	22.06.2011	A	Female	20.07.2020	faeces	Pos
		14.06.2017	A	Male	20.07.2020	faeces	Pos
38	The Netherlands	19.06.2016	A	Female	?	faeces	Neg
		17.06.2018	A	Male	?	faeces	Neg
39	United Kingdom	14.07.2012	A	Male	3.08.2020	faeces (pool)	Pos
		1.07.2007	A	Female	3.08.2020		
40	Ireland	21.07.2008	A	Female	21.07.2020	faeces	Neg
41	Spain	13.07.2008	A	Male	29.05.2020	faeces	Neg
42	Denmark	7.07.2008	A	Female	7.09.2020	faeces	Neg
43	Germany	4.07.2019	J		8.09.2020	faeces (pool)	Neg
		19.06.2018	A	Female	8.09.2020		
44	United Kingdom	3.07.2013	A	Female	7.09.2020	faeces	Neg
		16.06.2018	A	Male	7.09.2020	faeces	Neg
45	Belgium	21.06.2010	A	Female	14.09.2020	faeces	Pos
		16.07.2014	A	Female	14.09.2020	faeces	Pos
46	France	22.06.2018	A	Female	14.09.2020	faeces	Pos
		5.07.2019	J	Male	14.09.2020	faeces	Pos
47	United Kingdom	20.07.2018	A	Female	23.09.2020	faeces (pool)	Neg
		20.07.2018	A	Female	23.09.2020		
48	Austria	17.06.2016	A	Male	19.09.2020	faeces (pool)	Neg
		7.07.2008	A	Female	21.09.2020		
49	Germany	1Y	A	Male	23.09.2020	faeces (pool)	Neg
		19.06.2016	A	Male	23.09.2020		
50	Poland	2.07.2013	A	Male	29.09.2020	faeces	Neg
51	United Kingdom	3.07.2016	A	Female	12.10.2020	faeces	Pos
		3.07.2016	A	Female	12.10.2020	faeces	Pos
52	United Kingdom	3.07.2018	A	Male	28.10.2020	faeces	Neg
		30.06.2016	A	Female	28.10.2020	faeces	Neg
53	Germany	21.06.2014	A	Male	23.11.2020	faeces (pool)	Neg
		26.06.2015	A	Female	23.11.2020		
54	France	1.07.2012	A	Female	24.11.2020	faeces	Neg
55	Finland	24.06.2012	A	Female	15.,16.12.2020	faeces (pool)	Neg
		30.06.2015	A	Male	15.,16.12.2020		
56	France	14.07.2014	A	Female	21.12.2020	faeces (pool)	Neg
		22.06.2013	A	Male	21.12.2020		
57	France	17.06.2014	A	Female	18.02.2021	faeces	Neg
		11.06.2019	J	Male	18.02.2021	faeces	Neg
58	Italy	26.06.2011	A	Male	21.04.2021	faeces (pool)	Pos
		22.06.2019	J	Female	21.04.2021		
		10.07.2019	J	Female	21.04.2021		
59	Israel	16.06.2015	A	Female	25.04.2021	faeces	Neg
		4.07.2013	A	Female	24.04.2021	faeces	Neg
60	United Kingdom	1.07.2012	A	Male	22.,23.,24.5.2021	faeces	Neg
61	Hungary	3Y 10M at death	A	Male	NA	formalin fixed parafin embeded tissues	Pos

62	Sweden	16.07.2021	J	Female	6.02.2022	tissues	Neg
		6.07.2009	A	Male	7.03.2022	faeces (pool)	Neg
		19.06.2018	A	Female	7.03.2022		
63	Czech Republic	25.06.2021	J	Male	22.03.2022	faeces	Neg
		25.06.2021	J	Female	22.03.2022	faeces	Neg

NA: not applicable

?: unknown
